# A Novel Anti-Inflammatory Effect for High Density Lipoprotein

**DOI:** 10.1371/journal.pone.0144372

**Published:** 2015-12-17

**Authors:** Scott J. Cameron, Craig N. Morrell, Clare Bao, AnneMarie F. Swaim, Annabelle Rodriguez, Charles J. Lowenstein

**Affiliations:** 1 Departments of Medicine, Division of Cardiology, University of Rochester School of Medicine, Box 679, 601 Elmwood Avenue, Rochester, NY, 14652, United States of America; 2 Aab Cardiovascular Research Institute, University of Rochester School of Medicine, Box CVRI, 601 Elmwood Avenue, Rochester, NY, 14652, United States of America; 3 Department of Medicine, The Johns Hopkins University School of Medicine, 950 Ross Building, 720 Rutland Ave, Baltimore, MD, 21205, United States of America; 4 Department of Comparative Medicine, The Johns Hopkins University School of Medicine 733 N. Broadway, MRB 827, Baltimore, MD, 21205, United States of America; 5 Department of Cell Biology, University of Connecticut School of Medicine, E5050, 263 Farmington Avenue, Farmington, CT, 06030, United States of America; Emory University, UNITED STATES

## Abstract

High density lipoprotein has anti-inflammatory effects in addition to mediating reverse cholesterol transport. While many of the chronic anti-inflammatory effects of high density lipoprotein (HDL) are attributed to changes in cell adhesion molecules, little is known about acute signal transduction events elicited by HDL in endothelial cells. We now show that high density lipoprotein decreases endothelial cell exocytosis, the first step in leukocyte trafficking. ApoA-I, a major apolipoprotein of HDL, mediates inhibition of endothelial cell exocytosis by interacting with endothelial scavenger receptor-BI which triggers an intracellular protective signaling cascade involving protein kinase C (PKC). Other apolipoproteins within the HDL particle have only modest effects upon endothelial exocytosis. Using a human primary culture of endothelial cells and murine apo-AI knockout mice, we show that apo-AI prevents endothelial cell exocytosis which limits leukocyte recruitment. These data suggest that high density lipoprotein may inhibit diseases associated with vascular inflammation in part by blocking endothelial exocytosis.

## Introduction

HDL plays an important role in maintaining cholesterol homeostasis through the process of reverse cholesterol transport, mediating the centripetal movement of cholesterol from peripheral tissues to the liver and excretion into bile [1]. Clinical studies have shown that plasma levels of HDL and its major apolipoprotein component apoA-I are inversely related to cardiovascular events [2, 3]. Animal studies show that HDL and apoA-I are anti-atherogenic [4–6]. The major cardiovascular benefit of HDL was originally attributed to its role in one particular aspect of reverse cholesterol transport, transferring cholesterol from macrophages in atherosclerotic lesions to the liver [7, 8]. However, HDL also has anti-inflammatory properties that may further reduce the risk of cardiovascular events [3, 9–11]. HDL contains enzymes such as paraoxonase isoforms that metabolize lipid peroxides, decreasing oxidative stress [12], although some data suggests that paraoxonase does not protect LDL against oxidation *in vitro* [13]. HDL also decreases expression of endothelial adhesion molecules such as P-selectin, intercellular adhesion molecule-1 (ICAM-1), and vascular cell adhesion molecule (VCAM-1) through inhibition of sphingosine-1 phosphate signaling and nuclear factor kappaB (NF-kB) [14–16]. In addition, HDL inhibits expression of chemokines such as monocyte chemoattractant protein-1 (MCP-1) [17]. HDL binding to the SR-BI activates endothelial nitric oxide synthase and the production of NO that in turn decreases vascular inflammation [18–22]. HDL activates endothelial nitric oxide synthase (eNOS or NOS3) in part by delivery of mediators to endothelial cells, including estradiol, ceramide, and sphingosine-1 phosphate [23]. Finally, HDL signaling through SR-BI and its adaptor molecule PDZK1 promotes the integrity of the endothelial barrier.[24, 25]. However, diseases such as diabetes and the metabolic syndrome lead to alterations in HDL composition and function, decreasing the beneficial properties of HDL [3, 26–28]. Thus, normal HDL in healthy individuals has anti-oxidant and anti-inflammatory properties that may contribute to the beneficial effects of HDL upon the vasculature.

Exocytosis of granules called Weibel-Palade bodies containing pro-inflammatory and pro-thrombotic mediators leads to endothelial cells activation and vascular inflammation [22, 29]. HDL can be separated into HDL sub-fractions of varying density. Prior studies show that both HDL-2 and HDL-3 independently predict outcomes in coronary heart disease [30]. With the exception of one study, the majority of clinical outcome investigations show that both HDL-2 and HDL-3 protect against adverse vascular events [31]. We chose to study only HDL-3 rather than pure HDL with the aim of avoiding the potential complication of assigning a precise signaling pathway to a heterogenous population of HDL subfractions. At the start of this study, we chose to study only HDL-3 rather than pure HDL with the aim of avoiding the potential complication of assigning a precise signaling pathway to a heterogeneous population of HDL subfractions. Nonetheless, at the time of preparing this manuscript, a very recent study showed that HDL-3 rather than HDL-2 is responsible for the protective effects of HDL [32].

Since endothelial exocytosis contributes to thrombosis and myocardial infarct expansion [33, 34], we hypothesized that HDL-3 exerts its anti-inflammatory effects in part by altering a signal transduction pathway in endothelial cells which limits endothelial exocytosis of inflammatory and thrombotic granules. We show that HDL may prevent endothelial cell exocytosis through an interaction between apoA-I and the scavenger receptor BI (SR-BI). Our results suggest that decreased exocytosis mediates part of the anti-inflammatory effect of HDL.

## Material and Methods

### Materials

Human aortic endothelial cells (HAEC) and EGM-2 medium were obtained from Clonetics (Walkersville, MD). The promyelocytic leukemia cell line HL-60 was purchased from ATCC (Manassas, VA) and grown in Iscove's modified Dulbecco's medium with 4 mM L-glutamine adjusted to contain 1.5 g/L sodium bicarbonate, 80%; fetal bovine serum, 20%. All cells were cultured in a humidified environment supplemented with 95% air/5% CO_2_. All experimental procedures were conducted with HAEC at passages 3–5. Human thrombin was obtained from Sigma-Aldrich CO (St. Louis, MO) and stored as a 100 U/mL stock solution. For total protein staining of polyacrylamide gels, SimplyBlue Safestain was used (Invitrogen, Carlsbad, CA). Peroxynitrite and degraded peroxynitrite were obtained from Upstate Biotech (Lake Placid, NY). Rabbit IgG was obtained from Santa Cruz Biotechnology (Santa Cruz, CA). An antibody to apoA-I was from Novus Biologicals (Littleton, CO). The purified rabbit polyclonal antibody to SR-BI and the unpurified rabbit blocking antibody to SR-BI in rabbit serum were from Novus Biologicals (NB 400–104 and NB 400–113 respectively). Purified human apoA-II, apoC-I, and apoE were obtained from Biodesign (Saco, ME). Human HDL-3 (#80P-HD3-101) and apoA-I (#12P-105) was obtained from the Academy-Biomedical Co. (Houston, TX). 2’,7’-bis-(2-carboxyethyl)-5-(acetoxymethyl) ester (BCEF-AM) was obtained from Molecular Probes (Eugene, OR).

### Isolation of HDL from Human Plasma

**Fig 1:**
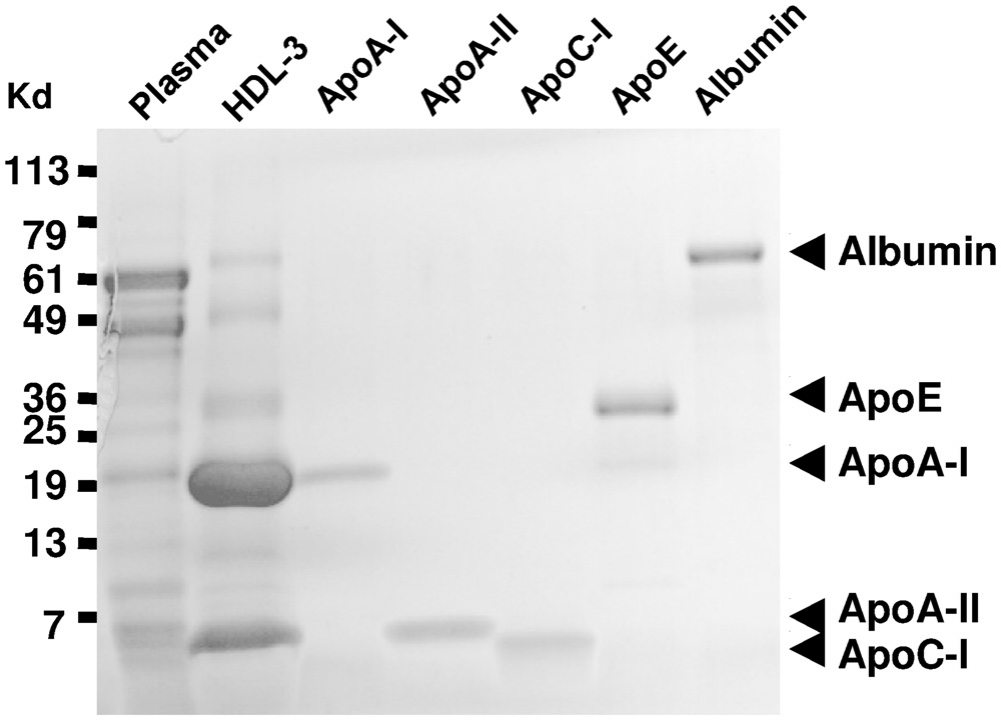
Lipoprotein electrophoresis. HDL-3 was isolated from the blood of a healthy human volunteer, fractionated by SDS-PAGE, and stained with Coomassie blue. Purified apolipoproteins were also loaded and fractionated as controls. The HDL-3 fraction contains a major apolipoprotein with the same relative mobility as ApoA-I.

Verbal consent was obtained from a self-initiated male volunteer for a 50mL one-time blood draw to isolate HDL-3 for in order to assess technical feasibility of a new project. Verbal consent was documented on page 45 of the project leader’s laboratory notebook. Neither IRB approval nor a waiver of permission was sought for the remainder of the study as no further donations were needed once it was clear HDL-3 was a viable signaling mediator. HDL-3 in purified form was thereafter purchased from a commercially-available vendor as noted under materials and methods. For the pilot studies in this investigation, the one-time blood draw was obtained from a fasted male individual and placed into EDTA tubes (containing 1.5 mg EDTA/ml) and mixed. Red blood cells were removed by centrifugation at 1000g for 15 mins at 10°C. The separated plasma was brought to a density of 1.12 g/L with solid potassium bromide, as described for sequential density ultracentrifugation to separate the apoB-containing apolipoproteins and HDL-2 subfraction from the higher density HDL subfraction [35]. The 1.12 g/L infranate was collected and brought to a density of 1.21 g/L, then ultracentrifuged at 100, 000g for 24 hours at 10°C. The 1.21 g/L supernatant containing the HDL subfraction was then centrifuged again under the same conditions for a minimum of 24 hours to concentrate the HDL subfraction and to remove any possibility of plasma protein contaminants. The concentrated HDL was dialyzed vs. normal saline containing 0.05% EDTA pH 7.4. The purity of the HDL fraction was confirmed by staining for total proteins using SimplyBlue solution, comparing this to human plasma and lipoprotein-deficient plasma from the same subjects, and purified apolipoproteins (Fig 1). HDL-3 was quantified by measuring the protein content using the Bradford assay, and found to be 5 mg/dL for the pilot study.

### Analysis of VWF Release from HAEC

Cells were grown to confluence in 24-well plates and deprived of serum 1 h prior to experimentation. Cells were pretreated with known concentrations of reagents for two hours in triplicate, washed, and stimulated with 1 U/mL thrombin in fresh serum-free EGM-2 medium for one hour. The extracellular medium was removed and VWF concentration was quantified using an ELISA from American Diagnostica (Stamford, CT).

### SDS-PAGE and Western Blotting

Cells were harvested in a Laemmli buffer (Bio-Rad, Hercules, CA) supplemented with β-mercaptoethanol. Protein at a concentration of 20 μg was loaded per well and separated by 10% SDS-PAGE. After overnight transfer to a nitrocellulose membrane using 20 V at 22°C (Bio-Rad equipment) and blocking in TBS-T (50 mM Tris, pH 8.0, 100 mM NaCl, 0.1% Tween 20) with 3% bovine serum albumin (room temperature for 1 h), the membrane was probed with primary antibody in 3% bovine serum albumin, TBS-T (room temperature for 2 h). After washing three times with TBS-T and secondary antibody exposure (anti-rabbit or anti-mouse IgG-horseradish peroxidase (1:1000, Bio-Rad) or anti-goat IgG-horseradish peroxidase (1:5000, Santa Cruz Biotechnology, Santa Cruz, CA) in 5% milk/TBS-T, (room temperature for 1 h), the membrane was washed three times with TBST-T, reacted with ECL reagent (PerkinElmer Life Sciences), and an image obtained by exposing the membrane to Kodak Biomax XAR Film (Eastman Kodak, Rochester, NY).

### Leukocyte Adhesion to HAEC *in vitro*

HL-60 cells were loaded with 5 μM BCEF-AM at 37°C for 20 minutes in calcium and magnesium-free HBSS solution without phenol red (Invitrogen, Grand Island, NY). The BCEF-loaded cells were washed three times with HBSS solution, and sequentially centrifuged at 1000 x g. HL-60 cells were loaded with 5 x10^7^ BCEF and then added to HAEC which had been preincubated for 2 hour in calcium and magnesium-free HBSS solution without phenol red ± 10^−4^ mg/mL apoA-I, and pretreated for 1 hour with 1 U/mL thrombin. The plate was covered with aluminum foil to prevent photo-bleaching and this was left at 4°C for 15 minutes to allow for cell adhesion. The HL-60/HAEC mixture was washed three times with fresh HBSS solution and fluoresce intensity was recorded in a multilabel counter (Wallac 1420; Perkin-Elmer, Boston, MA).

### Leukocyte Adhesion to HAEC *in vivo*

Wild-type C57BL6/J mice and apoA-I ^-/-^ counterparts were obtained from Jackson laboratories (Bar Harbor, ME). All mice were anesthetized with ketamine/xylazine and 0.05% rhodamine 6G (excitation: 528 nm; emission: 551 nm, Molecular Probes) was injected retro-orbitally and allowed to circulate for 15 mins to stain leukocytes *in vivo*. The animals were then prepared for intravital microscopy with an externalized mesentery. One mesenteric venule (approx. 100–200 μm in diameter) per animal was filmed for 5 mins min after a topical superfusion of 1 μM histamine to induce Weibel-Palade body exocytosis. Leukocyte adherence was expressed as the number of adhering fluorescent cells per square millimeter of venular surface, normalized to the diameter and length of segment viewed. Leukocyte rolling velocity for each time point was calculated by dividing the displacement vector per cell per unit time (n = 3), (10 ms interval, 30 ms capture/frame). Topical superfusion of 1 μM histamine induced Weibel-Palade body exocytosis and adhering platelets were manually counted.

### Cell Toxicity Assay

HAEC were grown to confluence in 96-well plates and serum-deprived for two hours in the presence of either 0.5 mg/dL HDL or the IC_50_ concentrations of apolipoproteins, obtained from the does-response experiments. After two hours, the Cell Titer 96 cell proliferation assay (Promega, Madison, WI) was used to assess formazan production at 490 nm as an index of cell viability, according to the manufacturer’s instructions.

### Statistical Analysis

Experimental conditions were compared using the Student’s t-test for comparisons between two groups or Analysis of Variance Between Groups (ANOVA) for comparisons between more than two groups. The null hypothesis was rejected when the p value was found to be less than 0.05.

## Results and Discussion

### HDL Inhibits Weibel-Palade Body Exocytosis

**Fig 2:**
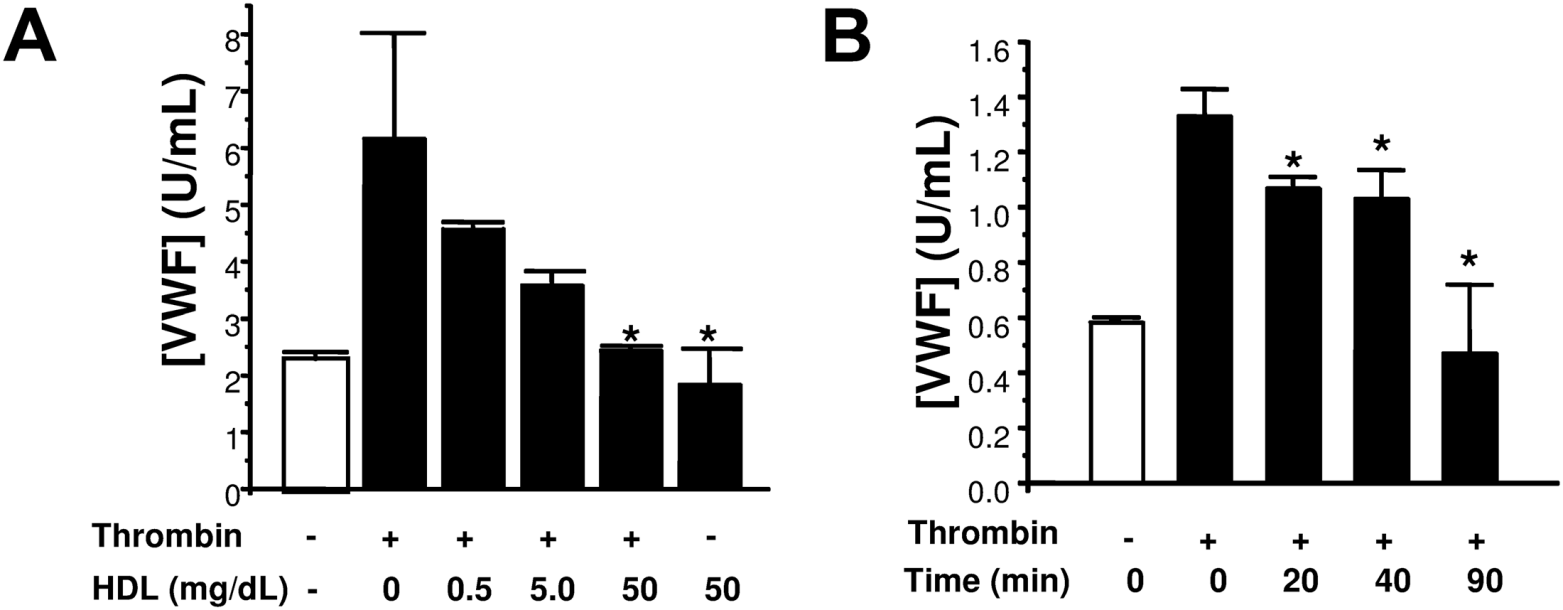
HDL decreases endothelial exocytosis. (A) Dose-response. Endothelial cells were pre-treated with various concentrations of purified HDL-3 within the human serum reference range for 2 h. (B). Time course. Endothelial cells were pre-treated with 0.5 mg/dL HDL-3 for 2 h. Cells were washed and stimulated with thrombin 1 U/ml, and the amount of VWF released over 1 h was measured by an ELISA (n = 3 ± S.D. *P < 0.05 vs. thrombin and 0 HDL-3).

To explore the effect of HDL upon endothelial exocytosis, we pre-treated human aortic endothelial cells (HAEC) with HDL-3, washed the cells, stimulated HAEC with thrombin, and measured the release of von Willebrand factor (VWF) as an indicator of exocytosis. Thrombin activates endothelial release of VWF (Fig 2A) and HDL-3 inhibits thrombin induced exocytosis in a dose-dependent manner (Fig 2A). HDL-3 inhibits exocytosis as early as 20 min after treatment, and HDL-3 inhibition progresses over 90 minutes (Fig 2B). These data suggest that HDL-3, or a component of HDL-3, inhibits endothelial Weibel-Palade body degranulation (endothelial exocytosis).

### Apolipoprotein A-I Inhibits Weibel-Palade Body Exocytosis

**Fig 3:**
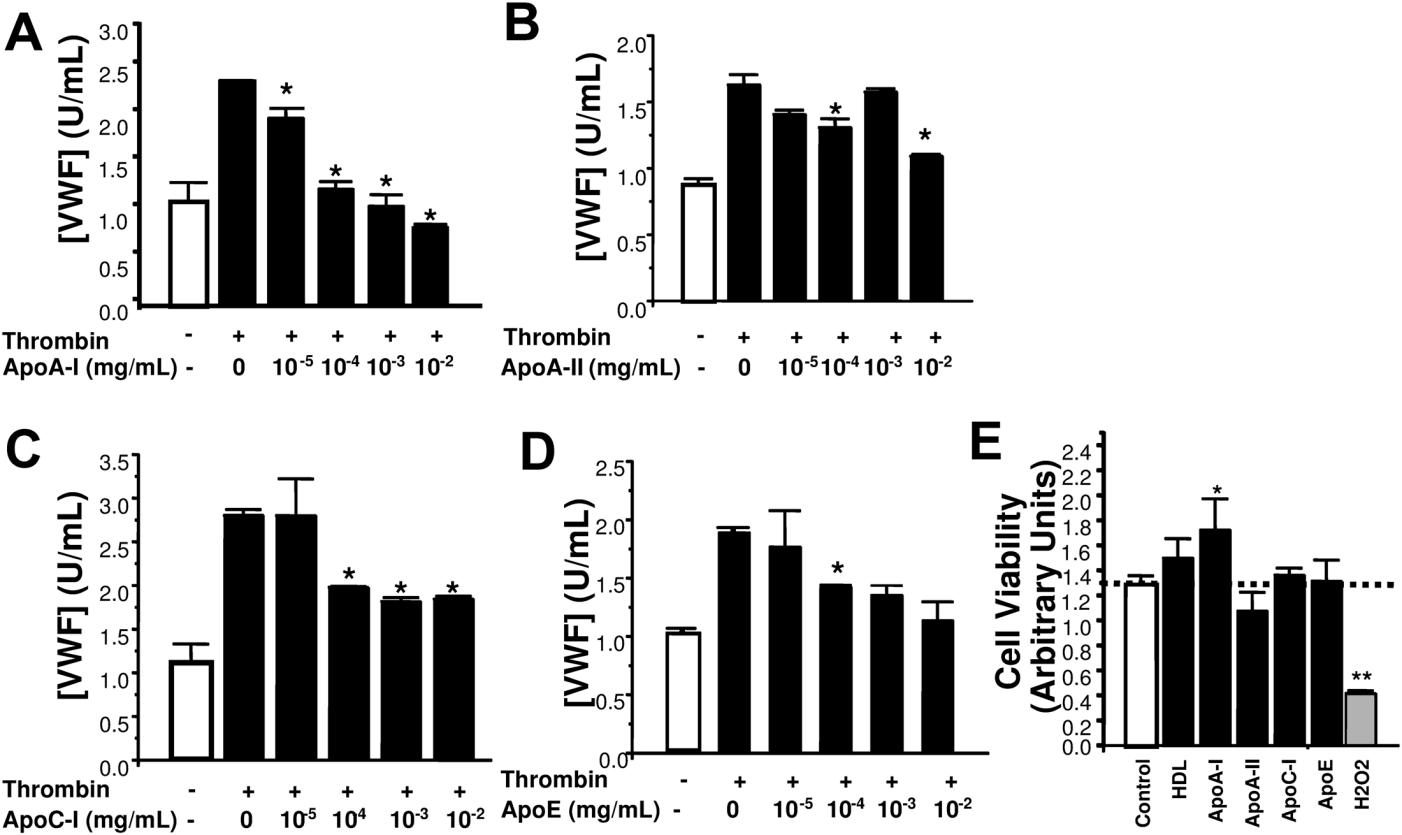
Purified apolipoproteins decrease endothelial exocytosis. (A-D) Dose-response for ApoA-I (human serum reference range is 0.75–1.75 mg/mL), and equivalent concentrations of Apo-AII, ApoC-I, and ApoE. Endothelial cells were pre-treated with various concentrations of purified apolipoproteins for 2 h. Cells were washed and stimulated with thrombin 1 U/ml, and the amount of VWF released over 1 h was measured by an ELISA (n = 3 ± S.D. *P < 0.05 vs. thrombin and 0 aplipoprotein). (E) Purified apolipoproteins are not cytotoxic to endothelial cells. Endothelial cells were pre-treated with IC_50%_ concentrations of purified apolipoproteins for 2 h. Hydrogen peroxide (H_2_O_2_) is a positive control for death. Cell viability was measured via formazan release with the MTS assay (n = 3 ± S.D. *P < 0.05 vs. control. **P < 0.01 vs. control.) The dashed line represents control levels.

In order to define the component of HDL that regulates exocytosis, we added purified apolipoproteins (apoA-I, apoA-II, apoC-I, and apoE) to endothelial cells for 2 hours, stimulated the cells with thrombin, and measured exocytosis. ApoA-I inhibits endothelial exocytosis in a dose-dependent manner with an IC_50%_ of between 10^−5^ to 10^−4^ mg/mL (Fig 3A). (The human serum reference range of apoA-I is 0.75–1.75 mg/mL; 10^−5^ corresponds to 0.36 nM.) Although apoA-II also inhibits exocytosis, a much higher concentration of apoA-II is required than apoA-I to achieve similar inhibition of exocytosis, with an approximate IC_50%_ value of 10^−2^ mg/mL (Fig 3B). ApoC-I inhibits endothelial exocytosis by approximately 40% at a dose of 10^−4^ mg/mL, and no further inhibition was observed using higher doses (Fig 3C). ApoE inhibits exocytosis of endothelial cells with an IC_50%_ between 10^−3^ and 10^−2^ mg/mL (Fig 3D). Purified apolipoproteins and HDL-3 are not toxic to endothelial cells at their IC_50%_ for inhibition of exocytosis (Fig 3E) and, in fact, apoA-I appears to promote cell viability even under resting conditions. These results suggest that apoA-I is a functionally significant component of HDL-3 that most potently inhibits endothelial exocytosis compared to the other apolipoproteins, and so we directed most of our remaining studies toward apo-AI.to the other apolipoproteins, and so we directed most of our remaining studies toward apo-AI.

### The Scavenger Receptor BI Mediates ApoA-I Inhibition of Exocytosis

**Fig 4:**
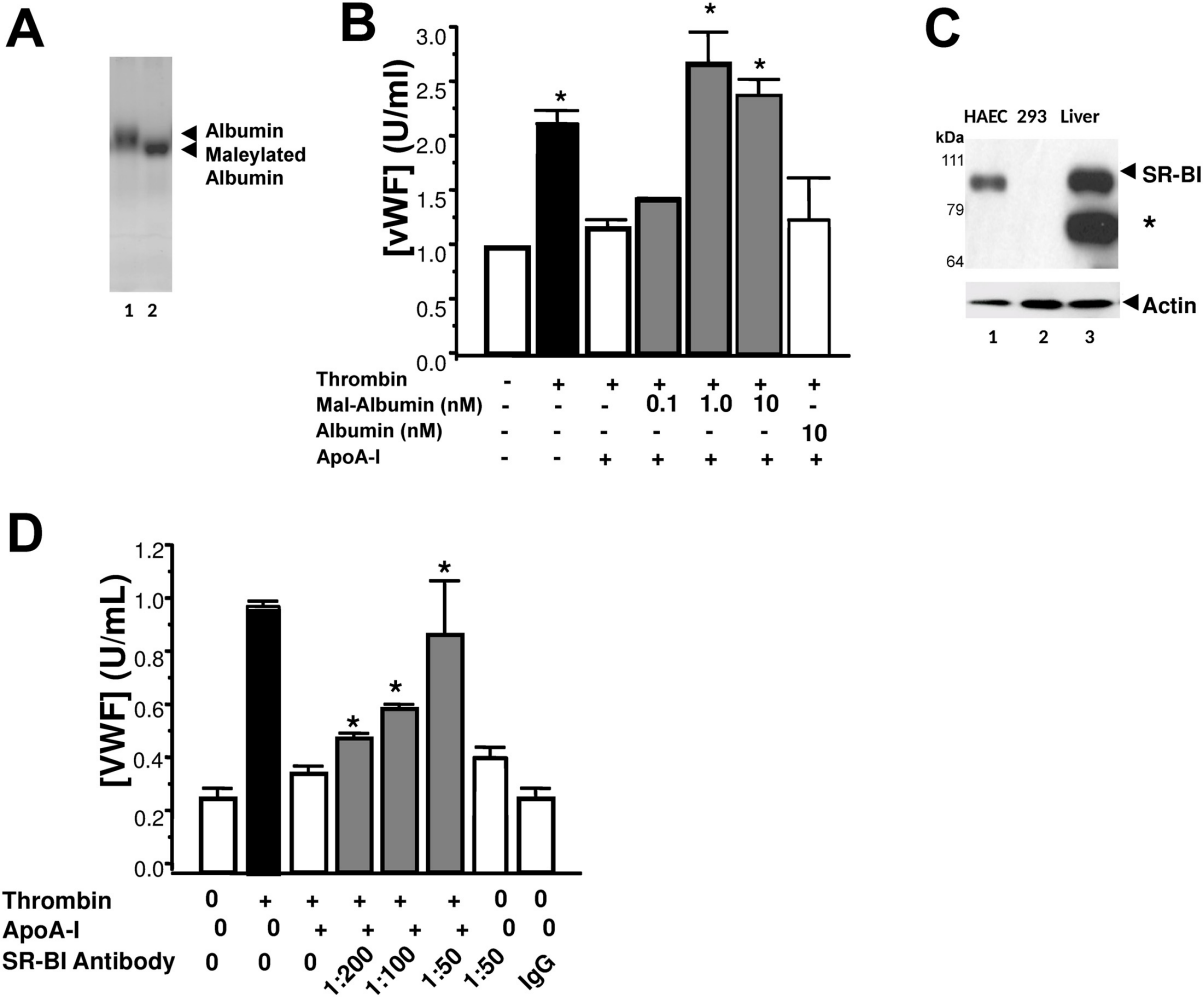
ApoA-I inhibits endothelial exocytosis via SR-BI. (A) Reaction of albumin (lane 1) with maleic acid (lane 2) leads to maleylated albumin with enhanced electrophoretic mobility, shown by Coomassie staining. (B) Maleylated albumin relieves the inhibition of endothelial exocytosis by ApoA-I. (C) SR-BI Expression: HAEC lysate, lane 1; Human Embryonic Kidney (HEK) 293 cell lysate, lane 2; wild-type mouse liver lysate, lane 3 were analyzed by immunoblotting with an SR-BI antibody. The signal at 82 kDa is consistent with the molecular weight of SR-BI; the band at 60 kDa is consistent with the molecular weight of unglycosylated SR-BI (asterisk). (D) SR-BI blocking antibody. Endothelial cells were pre-treated with an SR-BI blocking antibody, followed by the addition of 10^−4^ mg/mL apoA-I for 2 h. Cells were washed, stimulated with thrombin 1 U/ml, and the amount of VWF released over 1 h was measured by an ELISA (n = 3 ± S.D. *P < 0.05, vs. 0 apoA-I and 0 SR-BI receptor antibody).

HDL binds to the scavenger receptor BI (SR-BI) on endothelial cells, activating intracellular signaling cascades [18, 36, 37]. ApoA-I is a component of HDL that interacts with SR-BI [38, 39]. To investigate if apoA-I signals through SR-BI in endothelial cells, we synthesized maleylated albumin (mal-albumin), a molecule that clearly competitively antagonizes SR-BI-mediated ligand binding (Fig 4A) [39–42]. We found that maleylated albumin relieved the inhibitory effect of apoA-I on thrombin-stimulated endothelial cell exocytosis, using non-maleylated, native albumin as an internal control for consistency and assurance of endothelial cell viability (Fig 4B). Probing HAEC lysate clearly shows significant protein expression of SR-BI, enticing us to consider its functional significance (Fig 4C). To further confirm these results, we employed an alternate strategy: we added an SR-BI blocking antibody to HAEC, treated the cells with apoA-I for 2 h, stimulated the cells with thrombin, and measured VWF release into the extracellular medium. The blocking antibody to SR-BI decreases the apoA-I inhibition of exocytosis (Fig 4D). We also confirmed that endothelial cells express SR-BI (Fig 4D). These data suggest that apoA-I may interact with SR-BI, perhaps activating an intracellular signal transduction pathway that inhibits endothelial cell exocytosis, and therefore protects endothelial cells from certain noxious stimuli.

### ApoA-I attenuates Leukocyte Adhesion to Endothelial Cells *in Vitro*

**Fig 5:**
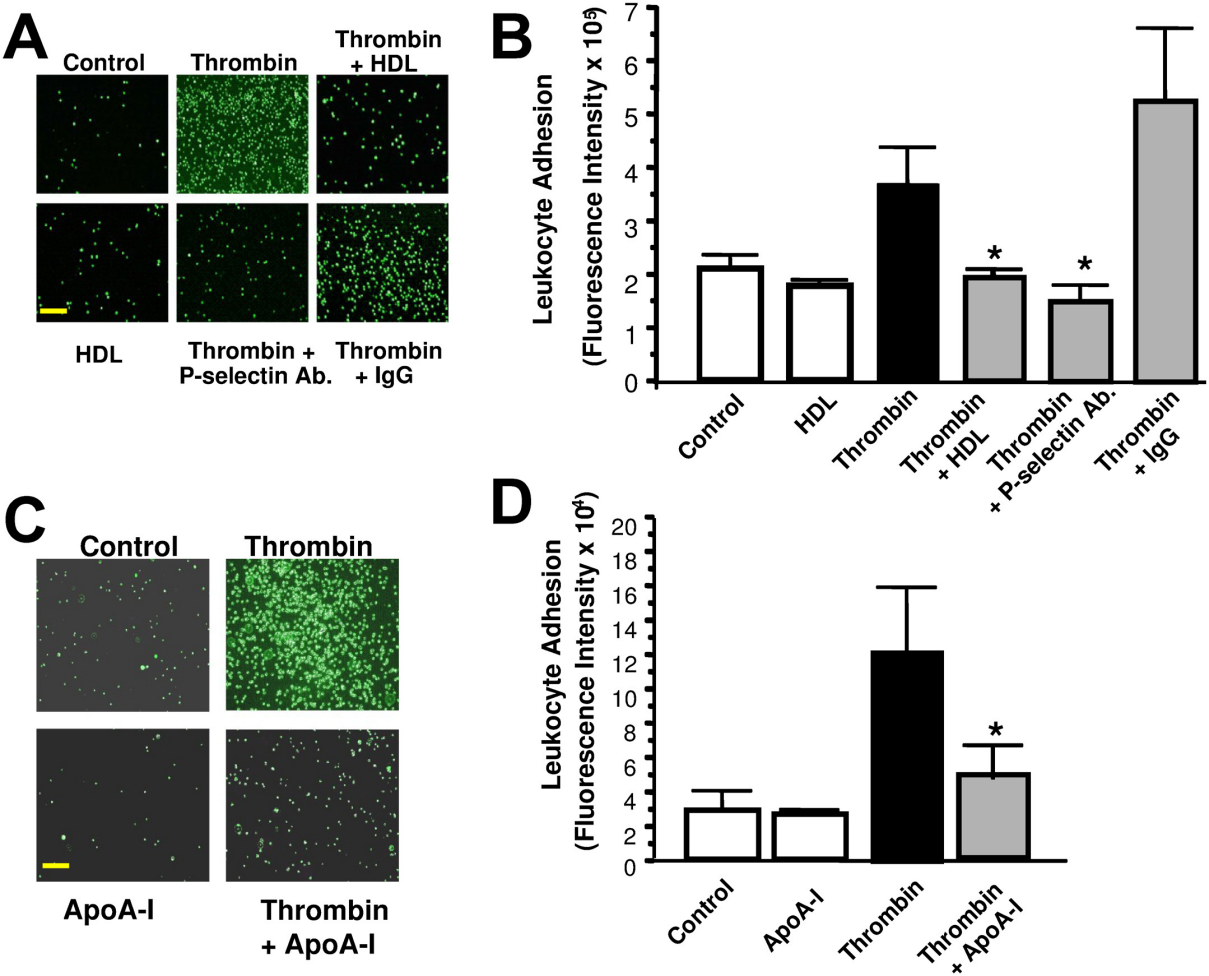
HDL and ApoA-I inhibit leukocyte adhesion to endothelial cells *in vitro*. (A-B) HDL inhibits p-selectin-mediated leukocyte adhesion to endothelial cells in vitro. Endothelial cells were pre-treated with 0.5 mg/dL HDL-3 for 2 h, washed, and stimulated with or without 1 U/mL thrombin for 1 h. Antibody to P-selectin or control IgG was added to some cells. (C-D). ApoA-I inhibition of leukocyte adherence. Endothelial cells were pre-treated with apoA-I 10^−4^ mg/ml for 2 h, washed, and stimulated with 1 U/mL thrombin for 1 h. The adherence of HL-60 cells labeled with BCEF-AM was measured as above, and imaged using a digital camera (n = 5 ± S.D. *P < 0.05, vs. thrombin only condition). Calibration bar is 50 μm.

We next explored the effect of HDL-3 and apoA-I on endothelial cell interactions with leukocytes as a surrogate model for endothelial-to-leukocyte recruitment, in part because endothelial-to-leukocyte recruitment is regulated by acute endothelial cell activation and granular secretion of adhesion molecules like p-selectin [43]. We pre-treated endothelial cells with buffer or HDL-3, added thrombin or media alone as a control, and then added human promyelocytic cells (HL-60) labeled with a fluorescent dye. We washed the cell mixture and measured the number of leukocytes adhering to endothelial cells. Thrombin increases leukocyte adherence to endothelial cells while prior incubation with an antibody to p-selectin prevented thrombin-induced leukocyte adherence to endothelial cells, demonstrating that this is an interaction mediated by P-selectin, a component of endothelial cell granules. Moreover, both purified HDL-3 and apo-AI decrease thrombin-stimulated leukocyte adhesion to endothelial cells (Fig 5). These data suggest that apoA-I inhibits endothelial exocytosis by preventing leukocyte adhesion through limiting p-selectin localization on the endothelial cell surface.

### Activated protein kinase C may play a role in apoA-I mediated regulation of endothelial exocytosis

**Fig 6:**
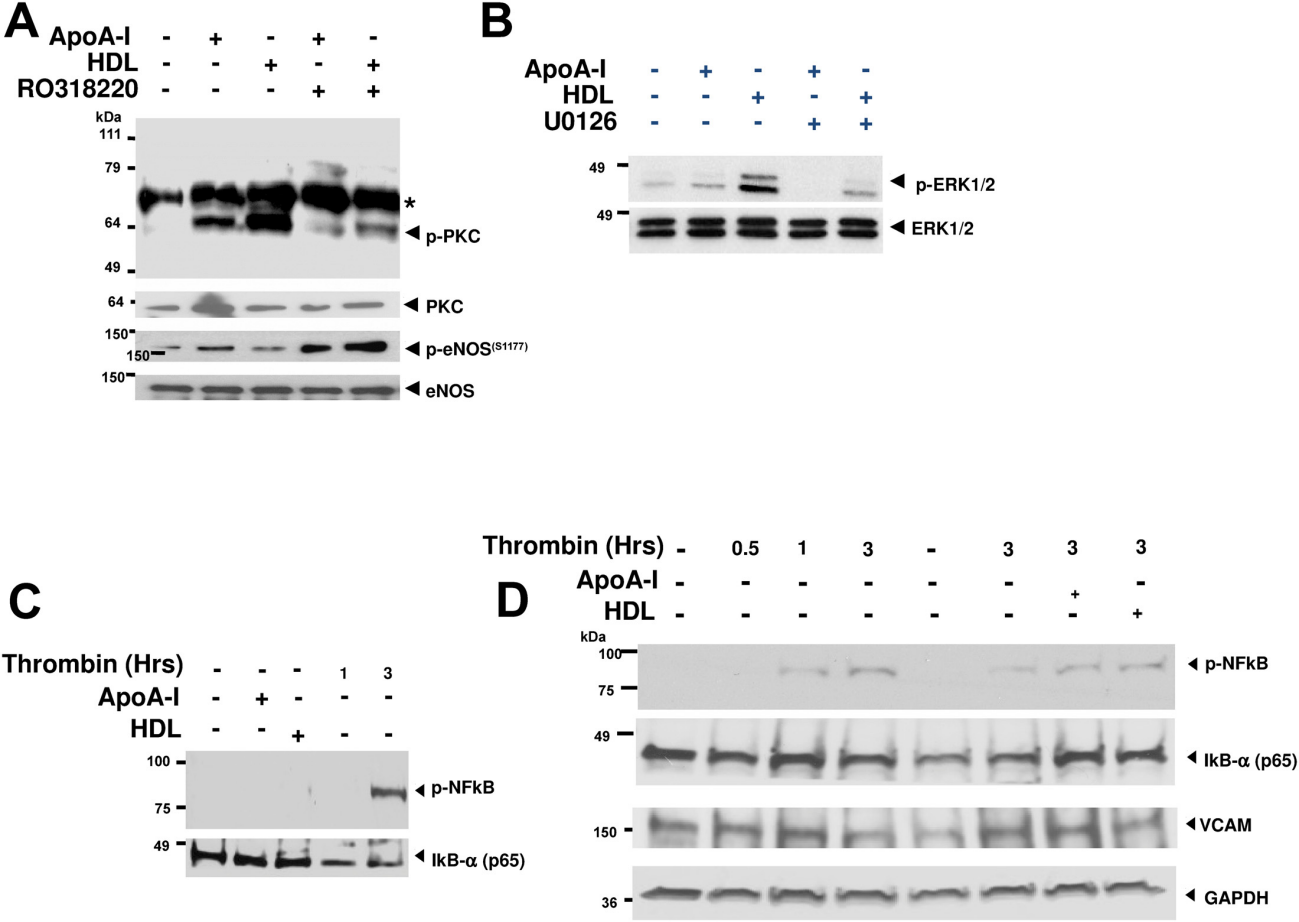
PKC mediates apoA-I inhibition of endothelial exocytosis. (A) HDL-3 and apoA-I activate PKC. Cells were treated with apoA-I (10^−4^ mg/mL) or HDL (0.5 mg/dL) and with or without the PKC inhibitor RO318220 for 2 h. PKC activation was assessed using a pan-phospho-PKC antibody (upper); asterisk indicates a non-specific band. eNOS activation was assessed using a phospho-eNOS (S1177)-specific antibody (akt phosphorylation site). (B) HDL and ApoA-I activate ERK1/2. Cells were pre-treated with or without the MEK inhibitor UO126 for 2 h, and then treated with apoA-I (10^−4^ mg/mL) or HDL (0.5 mg/dL) for 1 h. ERK activation was assessed using a phospho-ERK1/2 antibody (upper). This blots are representative of two others with similar results. (C) Cells treated with apoA-I (10^−4^ mg/mL), HDL-3 (0.5 mg/dL) for 1hr, or thrombin (1 U/mL) for 1 hr or 3hrs, and NFκB activation (p-NF κB) was assessed. Thrombin appears to activate NFkB (phospo-NFkB). (D) Treatment of endothelial cells with apoA-I (10^−4^ mg/mL) or HDL-3 (0.5 mg/dL) neither inhibits NFκB activation nor changes VCAM-1 expression following stimulation of cells with thrombin (1 U/mL) for 0.5–3 hrs. This suggests apoA-I and HDL-3 at the doses and in the time frame used do not exert an anti-inflammatory effect through these signaling pathways. Western blotting was conducted for phospo-NFkB, IkB-α (p65 subunit), vascular cell adhesion protein 1 (VCAM-1), or GAPDH. These blots are representative of two others with similar results.

SR-BI is a receptor in which signaling is reported to involve intracellular activation of: PKC, eNOS, and ERK1/2 [24]. As such, we used Western blotting to clarify the activation of these signaling pathways in HDL-3 and ApoA-I-stimulated endothelial cells. Stimulation of HAEC with HDL-3 or apoA-I activated PKC. PKC activation was attenuated by prior incubation of a PKC inhibitor, RO318220 (Fig 6A). We found that apoA-I but not HDL-3 was able to activate eNOS by phocphorylation on previously reported S1177 and T496 residues (Fig 6A and S1 Fig) [24]. HDL-3 also activated ERK-1/2 as previously reported (Fig 6B)[44]. We theorized that thrombin may also mediate endothelial cell inflammation through NFκB activation, though we found this requires around 3 hours of thrombin stimulation and neither HDL-3 nor apo-AI prior incubation were sufficient to block this effect. We also found that VCAM-1 protein expression was not changed following up to 3 hours of thrombin stimulation in HAEC (Fig 6C and 6D). While HDL-3 and apo-AI also weakly activate Pi3-K and downstream Akt in HAEC, we did not observe a consistent dependence upon PKC in activating these pathways in endothelial cells (S2 and S3 Figs). Furthermore, the weak activation of the akt pathway may in part explain why HDL-3 at the concentrations we use did not robustly lead to eNOS phosphorylation (Fig 6A and S1 Fig).

**Fig 7:**
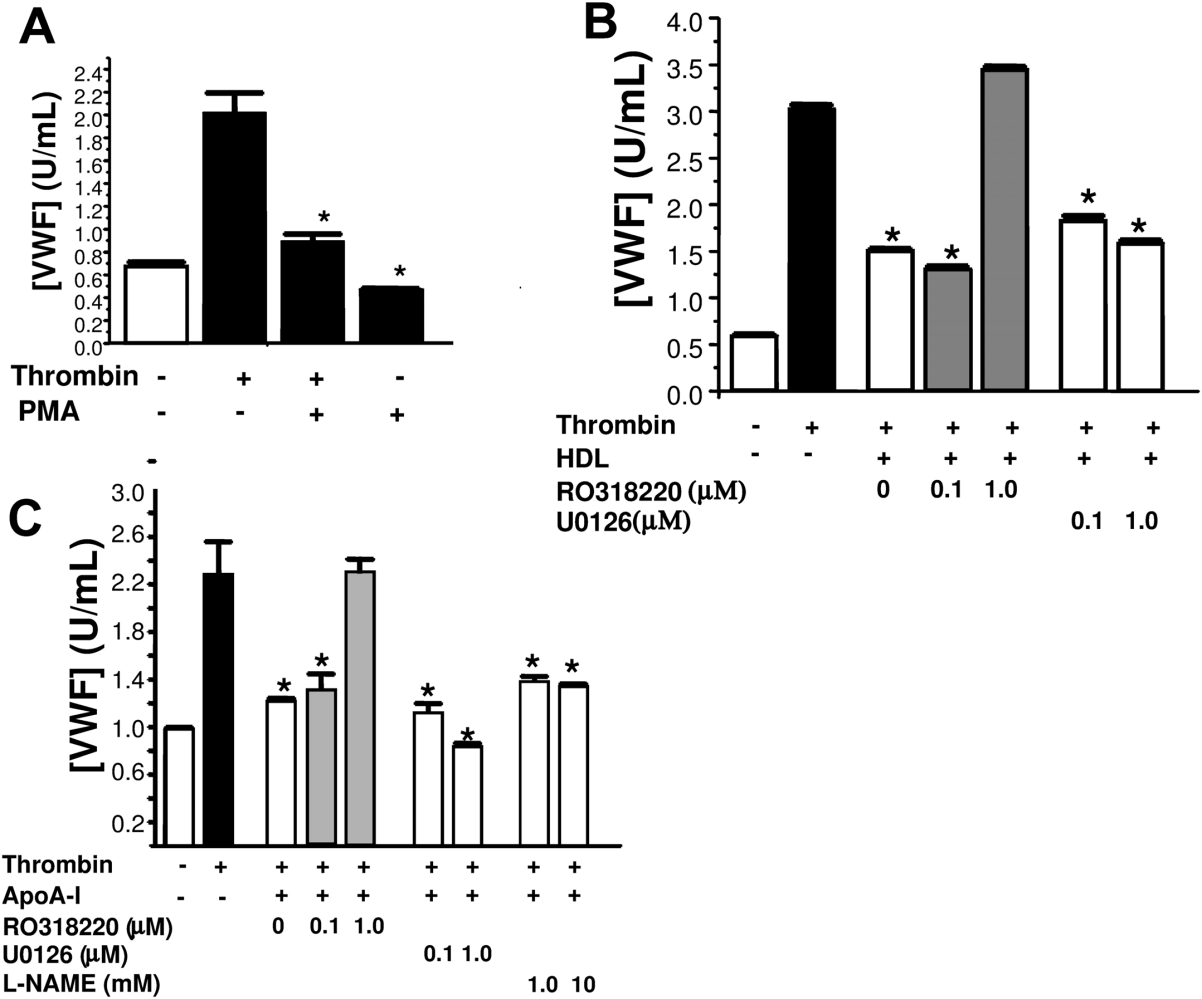
PKC mediates apoA-I inhibition of endothelial exocytosis. (A) PKC mediates apoA-I inhibition of endothelial exocytosis: PKC activation. Cells were pre-treated with the PKC activator PMA for 2 h. The cells were washed, then stimulated with thrombin for 1 h. The amount of VWF released over 1 h was measured by an ELISA (n = 3 ± S.D. *P < 0.05 for condition vs. thrombin alone). (B) Cells were pre-treated with the MAPKK inhibitor U0126, the PKC inhibitor RO318220 for 2 h. The cells were washed, treated with 0.5 mg/dL HDL-3 for 2 h, and then stimulated with thrombin for 1 h. The amount of VWF released over 1 h was measured by an ELISA (n = 3 ± S.D. *P < 0.05 for condition vs. thrombin + 0 HDL-3). (C) PKC mediates apoA-I inhibition of exocytosis. Cells were pre-treated with the MAPKK inhibitor U0126, the PKC inhibitor RO318220, or the NOS inhibitor L-NAME for 2 h. The cells were washed, treated with 0.5 mg/dL HDL-3 for 2 h, and then stimulated with thrombin for 1 h. The amount of VWF released over 1 h was measured by an ELISA (n = 3 ± S.D. *P < 0.05 for condition vs. thrombin + 0 apoA-I).

**Fig 8:**
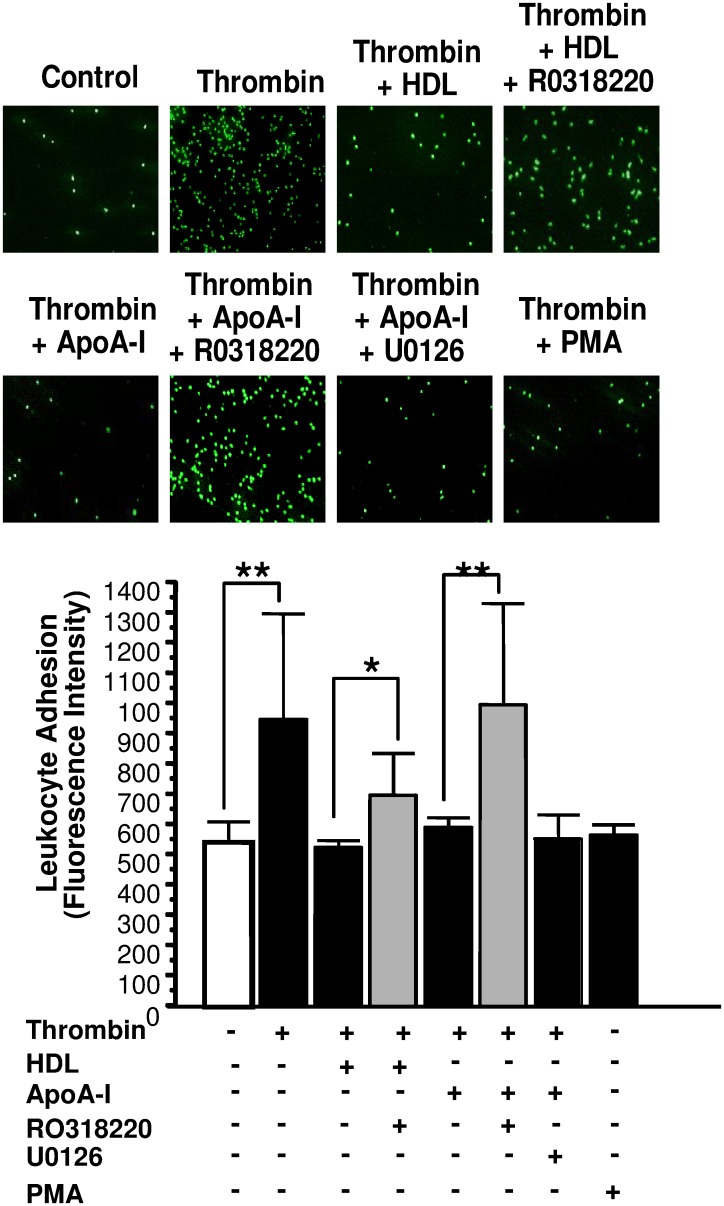
ApoA-I and HDL-3 inhibit leukocyte adhesion to endothelial cells in vitro via a PKC-mediated signaling pathway. Cells were pre-treated with the MAPKK inhibitor U0126 or the PKC inhibitor RO318220 for 2 h. The cells were then treated with apoA-I or HDL-3 for 1 h, and next stimulated with thrombin for 1 h. (A). The endothelial cells were incubated with HL-60 cells, washed and then imaged with a digital camera. (n = 5 ± S.D. *P < 0.05).

Stimulating HAEC with a PKC activator alone was sufficient to block thrombin-mediated degranulation of endothelial cells (Fig 7A), while selective inhibition of PKC in HAEC by RO318220 was able to the attenuate HDL-3-mediated blockade on endothelial cell degranulation, while inhibition of ERK1/2 and eNOS activation have little functional consequence upon endothelial cell exocytosis. Inhibition of PKC activation appeared to play a more critical role in relieving apoA-I-mediated blockade on endothelial cell degranulation subsequent to thrombin stimulation as reflected by VWF release (Fig 7B and 7C). Similarly, inhibition of PKC activation but not ERK1/2 activation in endothelial cells appeared to attenuate endothelial-to-leukocyte interaction in thrombin stimulated cells that have prior exposure to both HDLor apoA-I. This implies that endothelial cell surface p-selectin-mediated cell adhesion is important in this inflammatory process (Fig 8).

### Apo-AI regulates endothelial exocytosis acutely *in vivo*

**Fig 9:**
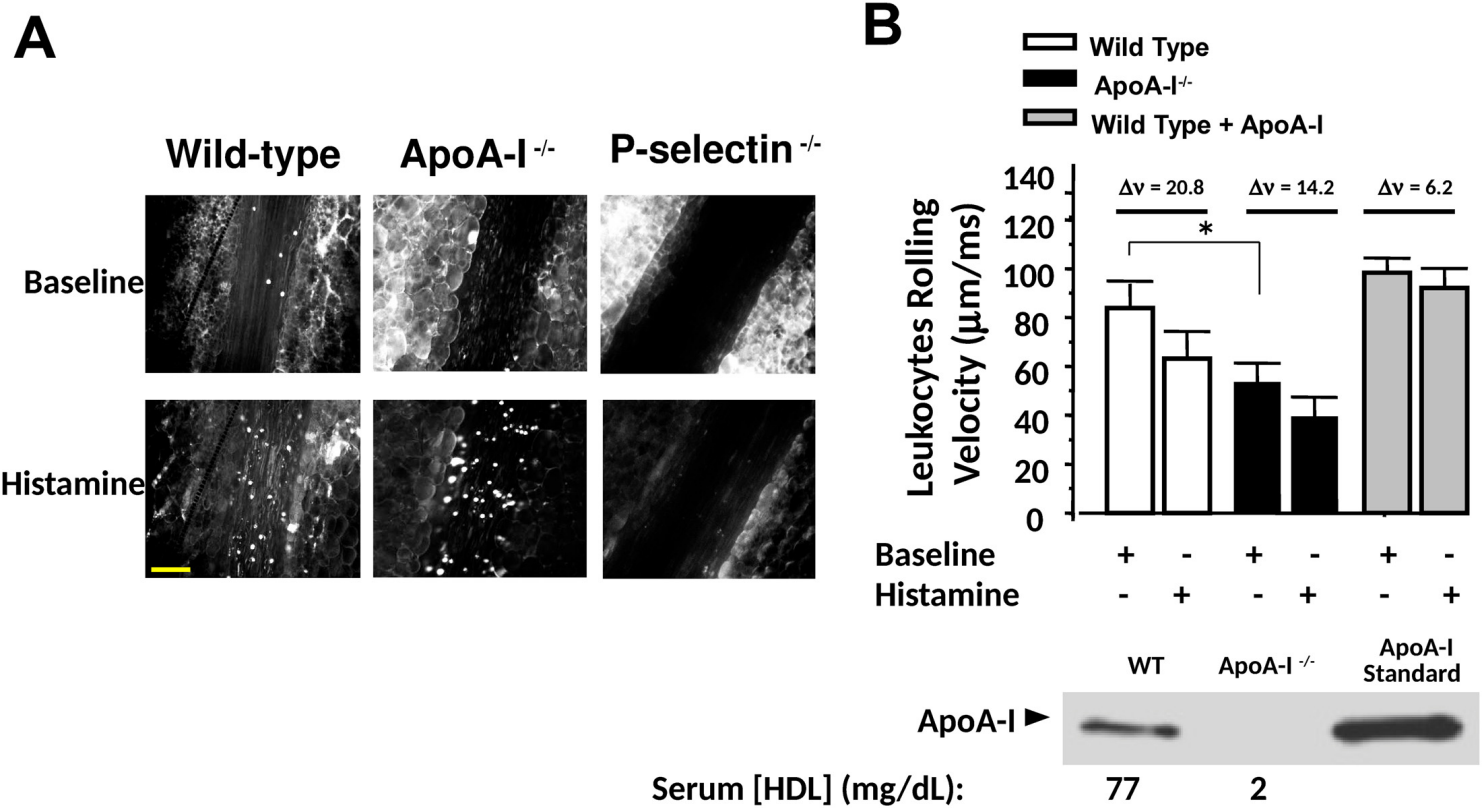
ApoA-I inhibits leukocyte adhesion to endothelial cells *in vivo*. (A) Venules from the small intestine were isolated in mice. Histamine (1μM) was superfused onto the venule and, after 5 mins, rhodamine 6G-labeled leukocytes were imaged using a digital fluorescence camera. Representative images are shown above. Calibration bar = 100 μm. P-selectin knockout mice (p-selectin^-/-^) do not display leukocyte-to-endothelial cell adhesion after histamine stimulation, validating this as an *in vivo* acute inflammatory adhesion assay. (B) ApoA-I knockout mice (apoA-I ^-/-^) have low plasma HDL concentration compared to WT mice and show increased adhesion of leukocytes to the vascular endothelium *in vivo* (decreased rolling velocity) compared to WT mice or apoA-I ^-/-^ mice injected with apo-AI (0.5mg, intraperitoneal) 24 hours before. Plasma HDL concentration in the apoA-I ^-/-^ mouse is noted below the blot. Changes in leukocyte rolling velocity (Δv, μm/ms) are noted above the graph for each treatment group. Western blotting was conducted for plasma apoAI (1:20 dilution) using an anti-apoA-I antibody. Leukocyte rolling velocity was assessed using 3 leukocytes per frame for n = 3–7 mice (mean rolling velocity ± SEM, *P < 0.05.

Leukocyte adhesion to the vascular endothelium *in vivo* was shown previously to be a p-selectin-mediated event [43]. Leukocytes were labeled in mice by injecting the fluorescent dye Rhodamine 6G, and dissecting videomicroscopy was employed to visualize leukocyte adhesion to the vascular endothelium before and for 5 minutes following endothelial cell activation with superfusion by 20 μl of the secretagogue histamine. Apo-AI knockout (apo-AI^-/-^) mice have almost no detectable plasma HDL compared to wild-type (WT) mice. We found that leukocyte-to-endothelial cell adhesion in response to histamine superfusion was augmented in apo-AI^-/-^ mice compared to WT mice, and the protective effect of apo-AI on endothelial inflammation was highlighted by showing decreased leukocyte adhesion following prior exogenous apo-AI injection into apo-AI^-/-^ mice. Furthermore, p-selectin knockout mice (p-selectin ^-/-^) do not show leukocyte-to-endothelial cell adhesion, showing specificity of this model in evaluating acute endothelial cell exocytosis (Fig 9). To assure correction of plasma apo-AI deficiency in apo-AI^-/-^ mice, peritoneal injection of mice with recombinant purified apo-AI time- and dose-dependently increased plasma apo-AI concentration as shown by Western blotting of plasma extracts (S3 Fig).

### Nitrated ApoA-I Fails to Inhibit Weibel-Palade Body Exocytosis

**Fig 10:**
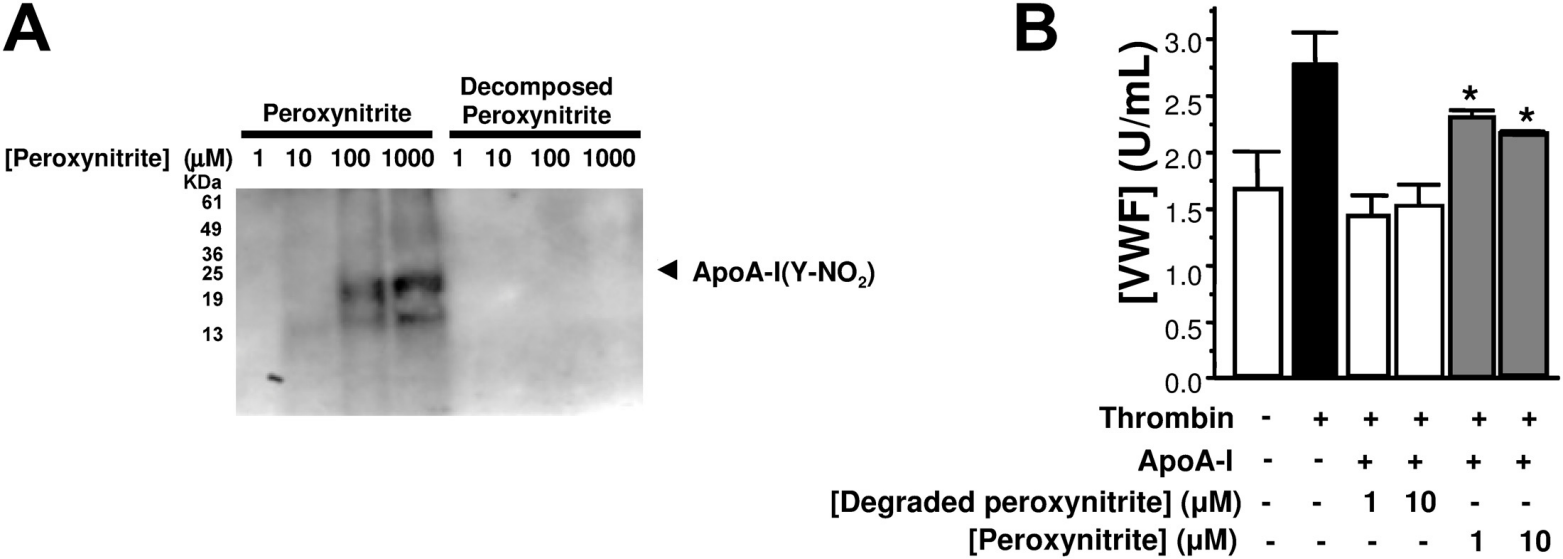
Nitration of apoA-I attenuates apoA-I inhibition of exocytosis. (A) Purified apoA-I (10^−4^ mg/mL) was incubated with increasing concentrations of peroxynitrite (left) or degraded peroxynitrite as a control (right). Mixtures were then fractionated by SDS-PAGE and immunoblotted with antibody to nitrotyrosine in order to assess tyrosine nitration of apoA-I. The position of apoA-I on the gel was confirmed with Coomassie staining (not shown). This experiment was repeated three times with similar results. (B). ApoA-I was treated with increasing concentrations of peroxynitrite or degraded peroxynitrite as a control. Endothelial cells were then incubated with treated or non-treated apoA-I 10^−4^ mg/mL for 2 h, washed, and stimulated with thrombin (1 U/ml). The amount of VWF released over 1 h was measured by an ELISA (n = 3 ± S.D. *P < 0.05, peroxynitrite vs. degraded peroxynitrite.

Dysfunctional HDL particles have decreased anti-inflammatory properties [3, 26]. Prior studies show that apoA-I is a target of myeloperoxidase-catalyzed nitration [45–48]. Furthermore, nitration of apoA-I impairs reverse cholesterol transport [45, 47]. We hypothesized that nitration of apoA-I limits its ability to block endothelial exocytosis. Accordingly, we compared the effect of native and nitrated apoA-I upon endothelial exocytosis. Incubation of apoA-I with peroxynitrite leads to nitration of tyrosine residues on apoA-I (Fig 10A). Nitration of apoA-I limits its ability to inhibit endothelial exocytosis by thrombin (Fig 10B). These data confirm that apoA-I inhibits endothelial exocytosis, and suggest that covalent modification of apoA-I amino acids, for example in certain inflammatory diseases, modulate its ability to regulate exocytosis.

## Discussion

The major finding of this study is that HDL-3 and, in particular, the apo-AI component of HDL-3, decreases leukocyte interactions with endothelial cells by inhibiting endothelial Weibel-Palade body exocytosis. We specifically show that HDL-3, a lipoprotein whose protective function is traditionally credited to reverse cholesterol transport chronically, also acts as an acute signaling mediator by ligating receptors on vascular endothelial cells and triggering a protective intracellular signaling. Our data therefore suggest a novel anti-inflammatory mechanism of HDL.

### HDL inhibits endothelial exocytosis

Endothelial exocytosis of Weibel-Palade bodies releases VWF and externalizes P-selectin. Our evidence suggests that HDL and its major apolipoprotein, apoA-I, inhibit exocytosis. HDL inhibition of exocytosis of VWF and P-selectin would reduce leukocyte and platelet interactions with endothelial cells, limiting inflammation. A major extension of the existing literature is that HDL-3 in our model initiates this protective anti-inflammatory signaling pathway acutely over minutes. HDL is reported to inhibit endothelial transcription of cytokines, chemokines, and intercellular adhesion molecules in response to stimuli such as CRP, IL-1ß, or TNF-a [15, 16, 49]. Our data confirm and further extend the work of others who show that HDL also inhibits a delayed (hours to days) transcriptional response to inflammatory stimuli, and suggests there may also be a more immediate effect of regulating intracellular endothelial cell granule mobilization. Thus, HDL exerts a dual effect on vascular inflammation, inhibiting both an immediate and a delayed endothelial response to injury.

### ApoA-I mediates the anti-exocytic effects of HDL

We identified apoA-I as a critical component of HDL-3 that inhibits endothelial exocytosis. Other HDL apolipoproteins also inhibit endothelial exocytosis in our hands, but at log-fold greater magnitude concentrations than that for apoA-I and without the same clear dose-dependence observed with apo-AI. These data support clinical observations that plasma apoA-I levels are negative predictors of atherosclerosis but apoA-II levels are not [50] Our data also support a set of important studies which show that peptides derived from apoA-I have anti-inflammatory effects *in vitro* and *in vivo* [51–54]. ApoA-I in our studies appears to inhibit exocytosis at lower concentrations than would be expected based on the amount of apoA-I in HDL-3. One possible explanation is that some of the domains of apoA-I that interact with SR-BI may be buried in the lipid milieu of HDL-3.

HDL-3 and apo-AI activate many signal transduction pathways acutely under our experimental conditions. Using the existing literature to guide us, we confirm in our studies that HDL-3 and apo-AI activate endothelial nitric oxide synthase, ERK1/2, Pi-3K, Akt, and PKC. Using Western blotting to confirm protein kinase phosphorylation and pharmacologic inhibitors for these pathways in functional assays, we show that acute exposure of endothelial cells to HDL-3 and apo-AI is regulated specifically rather than generally by endothelial PKC. While PMA activates additional signaling pathways downstream of PKC [55, 56], we show that PMA alone under our experimental conditions appears sufficient to promote endothelial cell activation, and PKC inhibition using RO318220 abrogates the protective effect of apo-AI upon endothelial exocytosis. We observe persistent blockade on endothelial exocytosis and inflammation by apo-AI in the presence of ERK-1/2 and nitric oxide synthase inhibitors, suggesting these latter two signaling pathways are less important in regulating endothelial cell inflammation acutely.

Consistent with prior studies, we show that apo-AI also activates endothelial nitric oxide synthase though this activation was weak and insufficient to regulate endothelial cell inflammation in our experimental conditions. In addition, while others report have reported apo-AI inhibits inflammatory NF-kB activation and expression of VCAM-1 in endothelial cells [9, 10, 20, 22, 42], we found that stimulation of endothelial cells with thrombin over minutes to hours does not significantly alter VCAM-1 expression, and thrombin-induced NF-kB activation was not inhibited by HDL-3 or apo-AI. These contrasting results more than likely reflect an acute effect of HDL-3 and apo-AI on exocytosis and inflammation over minutes compared to the well-known chronic protective effect of HDL-3 and apo-AI over hours to days. Chronic rather than acute apo-AI stimulation of endothelial cells likely requires cellular transcriptional activity. This assertion is further supported in our study using an apo-AI knockout mouse in which endothelial exocytosis and leukocyte recruitment over five minutes was augmented, then subsequently inhibited by injecting apo-AI back into these mice. Our studies thus build on the work of others, emphasizing that apoA-I has anti-inflammatory properties, though clearly acutely are well as chronically.

Although apoA-I blocks exocytosis in an SR-BI-dependent manner, our data do not necessarily demonstrate that free apoA-I protein binds to SR-BI in the absence of lipid. ApoA-I incorporated into lipids binds to SR-BI. ApoA-I added to our cell culture media may interact with lipid before signaling through SR-BI. In addition, non-apolipoprotein mediators of the HDL particle may be important cell signaling and the protective effects of HDL. For example, sphingosine-1-phosphate (S1P) receptor was demonstrated to be important for the signal transduction pathway by which HDL liberates NO from the vascular endothelium [23, 57]. For this reason and, due to the fact that our experimental conditions did not show robust eNOS activation by HDL-3, we focused more on apo-AI signaling events via SR-BI. However, our data show that apoA-I added to endothelial cells regulates exocytosis and inflammation in an SR-BI dependent fashion. These data are supported by studies of SR-BI conditional knockout mice which show that SR-BI plays an anti-atherogenic role not only in the liver and also in extrahepatic tissues as well [58].

### Nitration and ApoA-I

ApoA-I is a target of nitration catalyzed by myeloperoxidase (MPO) [46, 47] [59]. Nitration of tyrosine residues of ApoA-I diminishes cholesterol efflux from macrophages. We have found another effect of apoA-I nitration by observing nitrated ApoA-I cannot inhibit endothelial exocytosis as readily as native ApoA-I can. Our observations provide a possible mechanism explaining the observations of Zheng and colleagues, who found that nitration of apoA-I inhibits distinct beneficial properties of HDL [45, 46]. Since elevated levels of MPO predict the risk of myocardial infarction in patients with chest pain, increased endothelial exocytosis due to MPO catalyzed nitration of ApoA-I may provide a mechanistic link between MPO and acute coronary syndromes [60].

## Conclusions

Our data suggest an additional mechanism for the protective effects of HDL-3 in humans. In patients with stable coronary artery disease, plasma HDL concentration is inversely correlated with the risk of cardiovascular events. In patients with acute coronary artery syndromes, infusion of recombinant apoA-I causes the rapid regression of atherosclerotic lesions [61]. Our data suggest that a major role of apoA-I is to inhibit endothelial exocytosis acutely, thereby decreasing leukocyte and platelet trafficking in blood vessels that would otherwise cause vascular inflammation and thrombosis. It is, however, important to acknowledge that some studies have shown HDL can lose its protective effect and, in certain diseased conditions, anti-inflammatory HDL may be converted to a dysfunctional, pro-inflammatory HDL [62]. The biochemical triggers which convert protective HDL to dysfunctional HDL need further clarification. Our observations suggest that an additional protective effect of HD—and specifically the apo-AI component of HDL-3—upon the vasculature may be due in part to a decrease in endothelial exocytosis.

## Supporting Information

S1 Fig Alternative ways ApoA-I and HDL-3 can activate eNOS. Purified Endothelial cells were stimulated with apoA-I (10^−4^ mg/mL) or HDL-3 (0.5 mg/dL) for 1 hr. ApoA-I but not HDL-3 activates endothelial eNOS by threonine 496 phosphorylation (T496). Samples were immunoblotted with a phospho-eNOS (T496) antibody. The blot was reprobed with a total eNOS antibody as a loading control. Data are representative of blots from two additional experiments with similar results.
(TIFF)

S2 Fig PKC does not alter HDL-3 and ApoA-I signaling through Akt. Purified Endothelial cells were incubated with R0318220 (1 μM) for 1 hr, then stimulated with apoA-I (10^−4^ mg/mL) or HDL-3 (0.5 mg/dL) for 1 hr, or stimulated with thrombin alone (1 U/mL, 3 hrs). Both HDL and ApoA-I activate endothelial akt (p-Akt) which is not altered by prior treatment of endothelial cells with the PKC inhibitor, R0318220, at the dose used in our experiments. Samples were immunoblotted with a phospho-Akt antibody to show activated Akt. The blot was reprobed with a total Akt and a GAPDH antibody as loading controls. Data are representative of blots from two additional experiments with similar results.
(TIFF)

S3 Fig PKC alters ApoA-I signaling through PI3K. Purified Endothelial cells were incubated with R0318220 (1 μM) for 1 hr with or without apoA-I (10^−4^ mg/mL) or HDL-3 (0.5 mg/dL) for 1 hr, or stimulated with thrombin alone (1 U/mL, 3 hrs). ApoA-I but not HDL-3 activates endothelial PI3K (p-PI3K), and this is inhibited by prior incubation of the PKC inhibitor, R0318220. Activated PI3K therefore may be an additional activated signaling pathway which explains the greater anti-inflammatory effect of ApoA-I compared to HDL-3 on human endothelial cells. ApoA-I or HDL-3 do not affect the activation of NFKB (p-NFKB) in the presence or absence of the PKC inhibitor, R0318220. Thrombin stimulation (1 U/mL, 3 hrs) was used as a positive control for NFKB activation. Samples were immunoblotted with a phospho-PI3K antibody or a phospho-NFKB antibody to show activated PI3K or NFKB, respectively. The blot also probed with a total PI3K, a IkB-α (p65 subunit) antibody, or a GAPDH antibody as loading controls. Data are representative of blots from two additional experiments with similar results.
(TIFF)

S4 Fig Exogenous apoA-I injection. Mice were injected intraperitoneally with human apoA-I at the concentrations and time points shown. A blood sample was drawn and serum was isolated for apoA-I. 1 μL of mouse serum was diluted 1:20 before SDS-PAGE.
(TIFF)
