# Supplementary figures and images for: A Novel Anti-Inflammatory Effect for High Density Lipoprotein
Source: PLoS One. 2015 Dec 17;10(12):e0144372. doi: 10.1371/journal.pone.0144372 (PMC4683005; doi:10.1371/journal.pone.0144372)

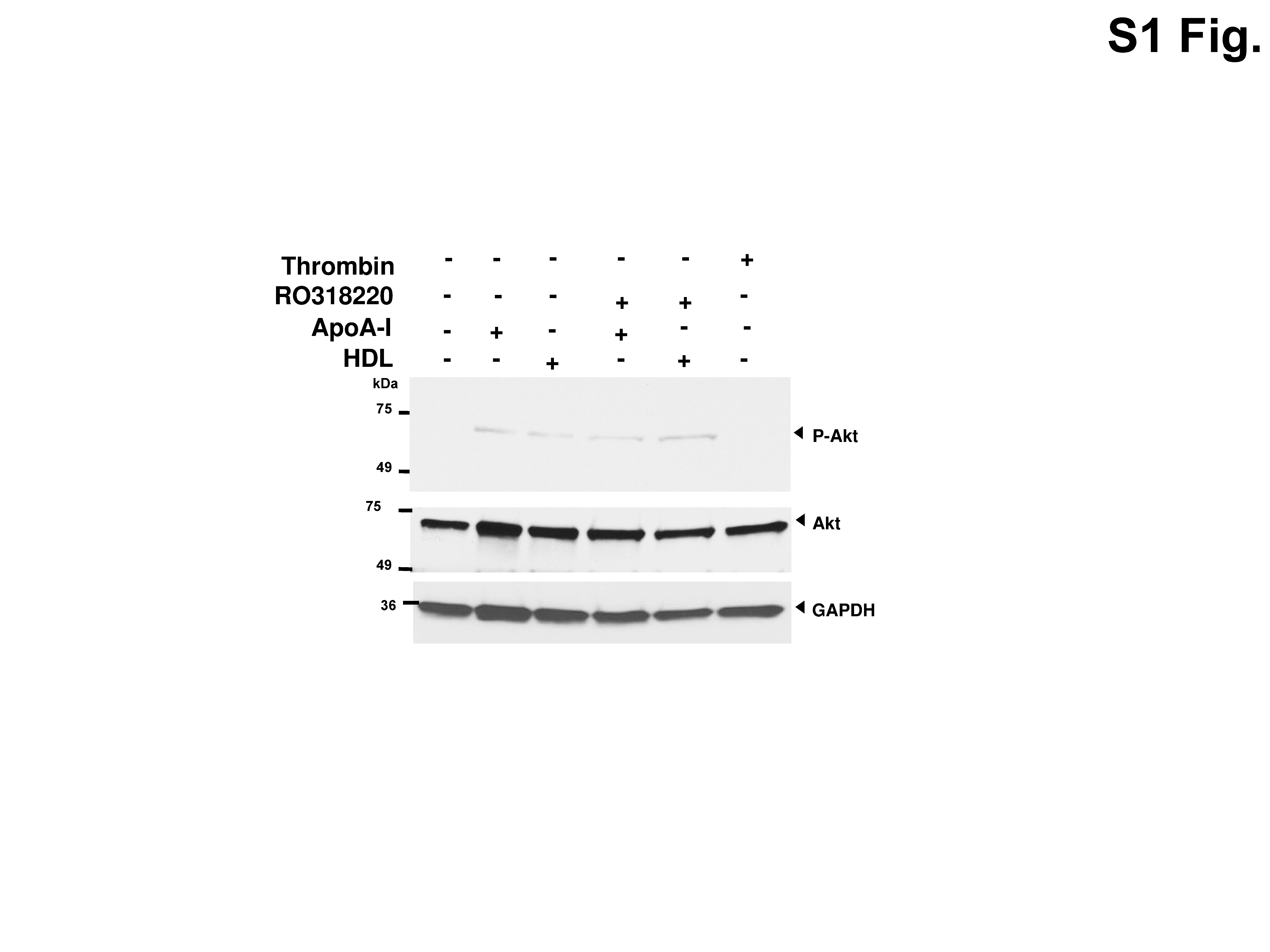

Supplement: S1 Fig — Purified Endothelial cells were stimulated with apoA-I (10−4 mg/mL) or HDL-3 (0.5 mg/dL) for 1 hr. ApoA-I but not HDL-3 activates endothelial eNOS by threonine 496 phosphorylation (T496). Samples were immunoblotted with a phospho-eNOS (T496) antibody. The blot was reprobed with a total eNOS antibody as a loading control. Data are representative of blots from two additional experiments with similar results. (TIFF) [file pone.0144372.s001.tiff]

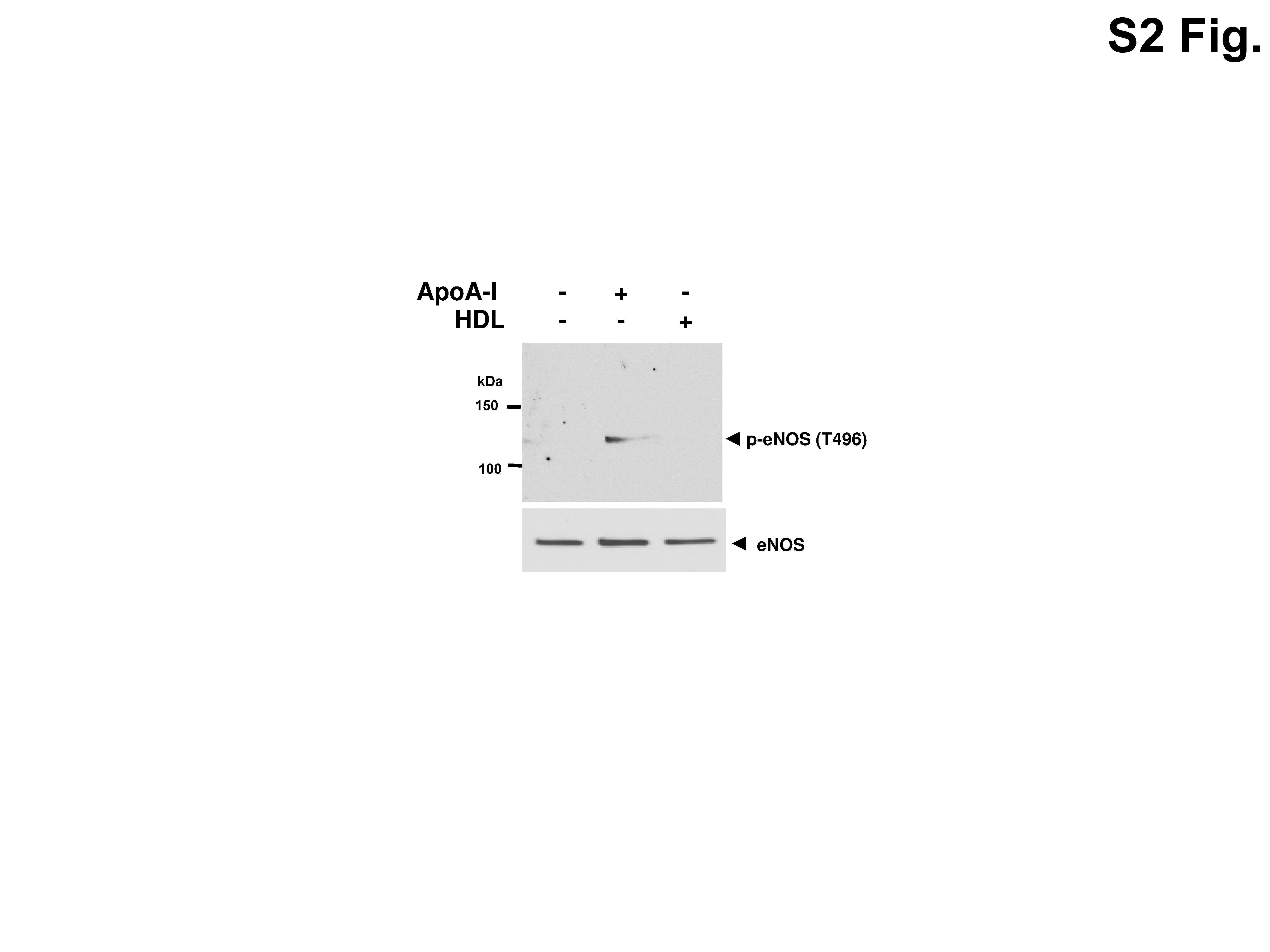

Supplement: S2 Fig — Purified Endothelial cells were incubated with R0318220 (1 μM) for 1 hr, then stimulated with apoA-I (10−4 mg/mL) or HDL-3 (0.5 mg/dL) for 1 hr, or stimulated with thrombin alone (1 U/mL, 3 hrs). Both HDL and ApoA-I activate endothelial akt (p-Akt) which is not altered by prior treatment of endothelial cells with the PKC inhibitor, R0318220, at the dose used in our experiments. Samples were immunoblotted with a phospho-Akt antibody to show activated Akt. The blot was reprobed with a total Akt and a GAPDH antibody as loading controls. Data are representative of blots from two additional experiments with similar results. (TIFF) [file pone.0144372.s002.tiff]

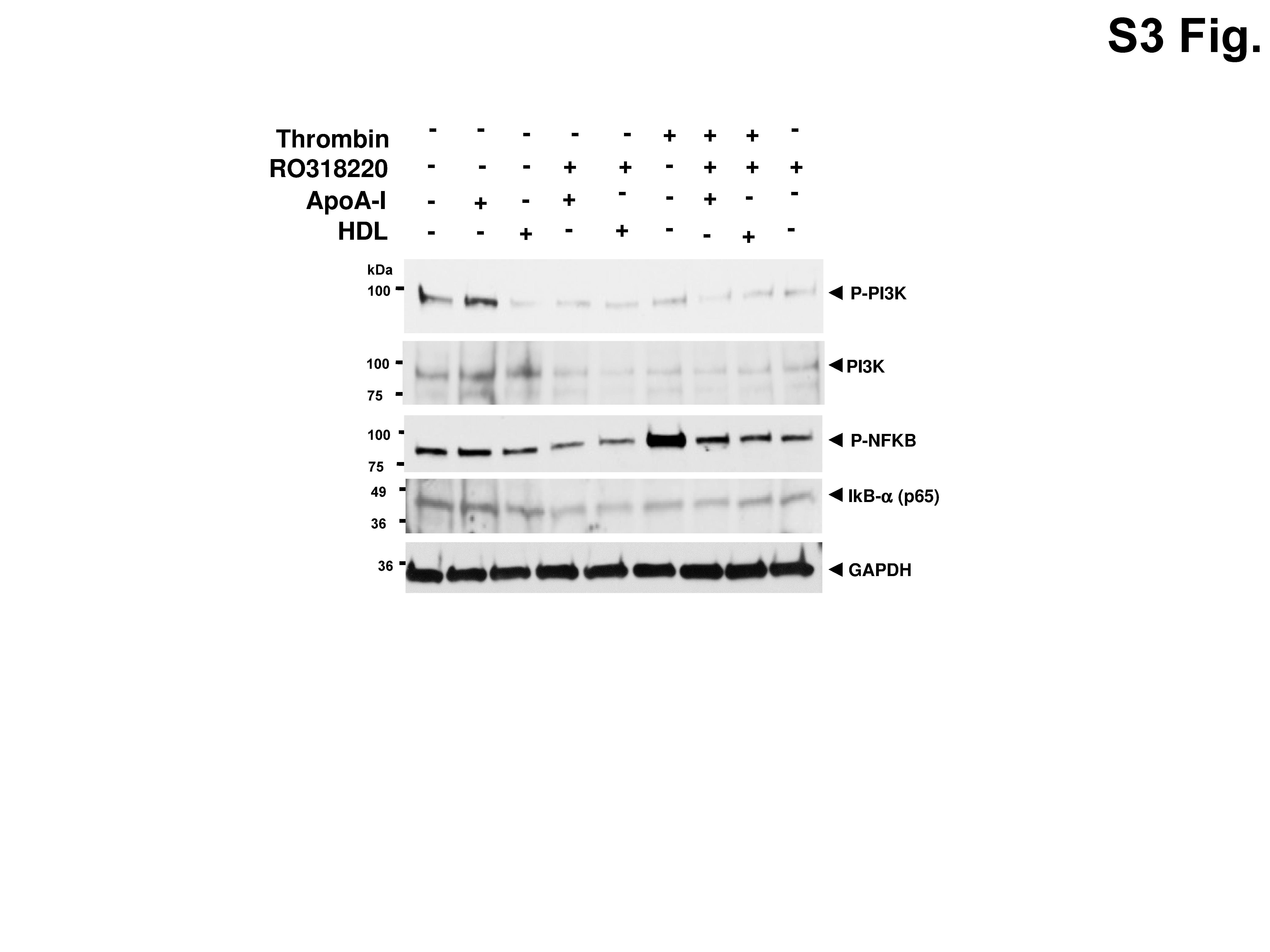

Supplement: S3 Fig — Purified Endothelial cells were incubated with R0318220 (1 μM) for 1 hr with or without apoA-I (10−4 mg/mL) or HDL-3 (0.5 mg/dL) for 1 hr, or stimulated with thrombin alone (1 U/mL, 3 hrs). ApoA-I but not HDL-3 activates endothelial PI3K (p-PI3K), and this is inhibited by prior incubation of the PKC inhibitor, R0318220. Activated PI3K therefore may be an additional activated signaling pathway which explains the greater anti-inflammatory effect of ApoA-I compared to HDL-3 on human endothelial cells. ApoA-I or HDL-3 do not affect the activation of NFKB (p-NFKB) in the presence or absence of the PKC inhibitor, R0318220. Thrombin stimulation (1 U/mL, 3 hrs) was used as a positive control for NFKB activation. Samples were immunoblotted with a phospho-PI3K antibody or a phospho-NFKB antibody to show activated PI3K or NFKB, respectively. The blot also probed with a total PI3K, a IkB-α (p65 subunit) antibody, or a GAPDH antibody as loading controls. Data are representative of blots from two additional experiments with similar results. (TIFF) [file pone.0144372.s003.tiff]

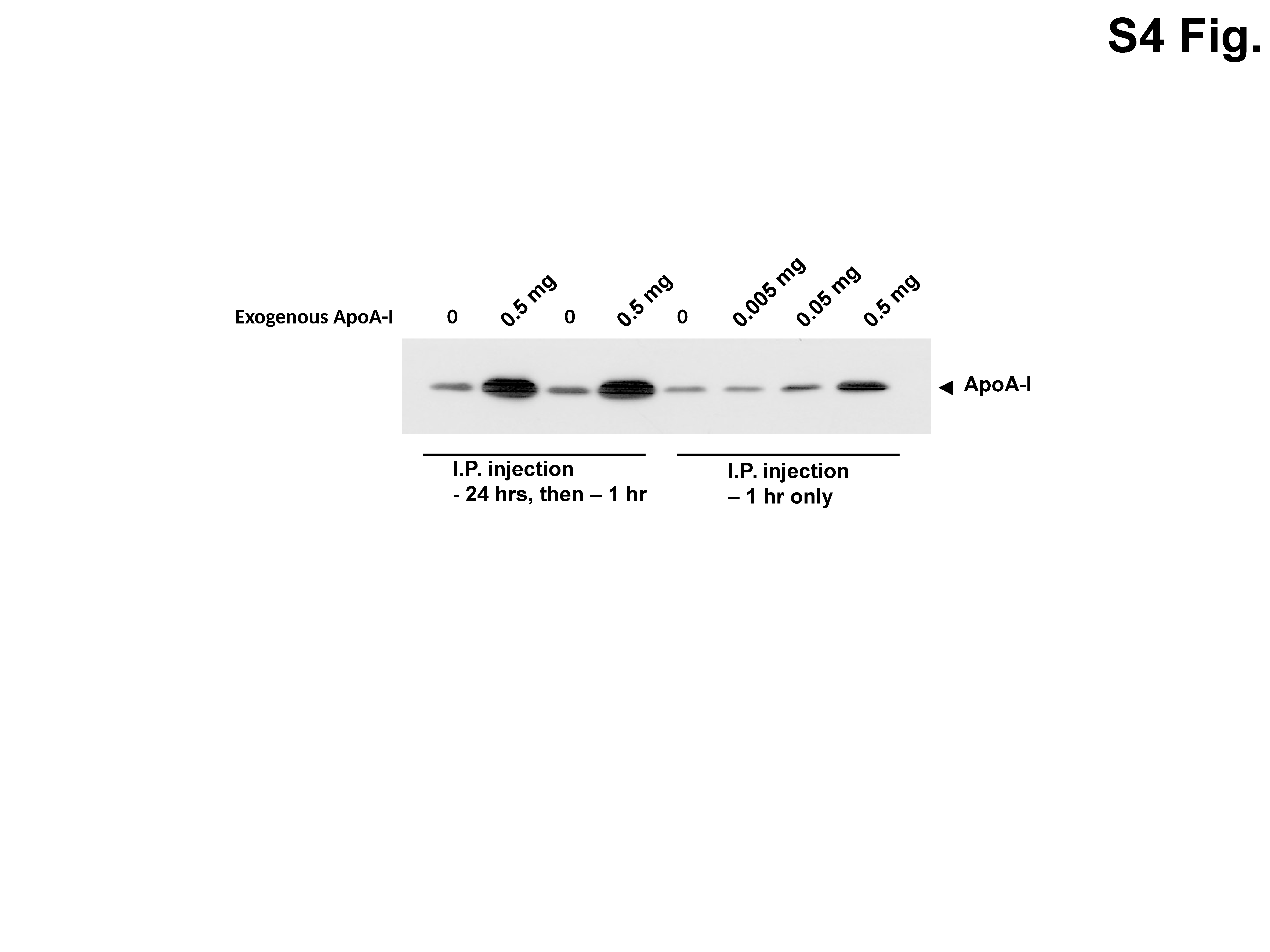

Supplement: S4 Fig — Mice were injected intraperitoneally with human apoA-I at the concentrations and time points shown. A blood sample was drawn and serum was isolated for apoA-I. 1 μL of mouse serum was diluted 1:20 before SDS-PAGE. (TIFF) [file pone.0144372.s004.tiff]
